# Quantitative Analysis of Vortical Blood Flow in the Thoracic Aorta Using 4D Phase Contrast MRI

**DOI:** 10.1371/journal.pone.0139025

**Published:** 2015-09-29

**Authors:** Jochen von Spiczak, Gerard Crelier, Daniel Giese, Sebastian Kozerke, David Maintz, Alexander Christian Bunck

**Affiliations:** 1 Department of Radiology and Neuroradiology, University Hospital of Cologne, Cologne, Germany; 2 Institute for Biomedical Engineering, University and ETH Zurich, Zurich, Switzerland; COLORADO STATE UNIVERSITY, UNITED STATES

## Abstract

**Introduction:**

Phase contrast MRI allows for the examination of complex hemodynamics in the heart and adjacent great vessels. Vortex flow patterns seem to play an important role in certain vascular pathologies. We propose two- and three-dimensional metrics for the objective quantification of aortic vortex blood flow in 4D phase contrast MRI.

**Materials and Methods:**

For two-dimensional vorticity assessment, a standardized set of 6 regions-of-interest (ROIs) was defined throughout the course of the aorta. For each ROI, a heatmap of time-resolved vorticity values $\vec{\omega }=\nabla \vec{v}$ was computed. Evolution of minimum, maximum, and average values as well as opposing rotational flow components were analyzed. For three-dimensional analysis, vortex core detection was implemented combining the predictor-corrector method with λ_2_ correction. Strength, elongation, and radial expansion of the detected vortex core were recorded over time. All methods were applied to 4D flow MRI datasets of 9 healthy subjects, 2 patients with mildly dilated aorta, and 1 patient with aortic aneurysm.

**Results:**

Vorticity quantification in the 6 standardized ROIs enabled the description of physiological vortex flow in the healthy aorta. Helical flow developed early in the ascending aorta (absolute vorticity = 166.4±86.4 s^-1^ at 12% of cardiac cycle) followed by maximum values in mid-systole in the aortic arch (240.1±45.2 s^-1^ at 16%). Strength, elongation, and radial expansion of 3D vortex cores escalated in early systole, reaching a peak in mid systole (strength = 241.2±30.7 s^-1^ at 17%, elongation = 65.1±34.6 mm at 18%, expansion = 80.1±48.8 mm^2^ at 20%), before all three parameters similarly decreased to overall low values in diastole. Flow patterns were considerably altered in patient data: Vortex flow developed late in mid/end-systole close to the aortic bulb and no physiological helix was found in the aortic arch.

**Conclusions:**

We have introduced objective measures for quantification of vortical flow in 4D phase contrast MRI. Vortex blood flow in the thoracic aorta could be consistently described in all healthy volunteers. In patient data, pathologically altered vortex flow was observed.

## Introduction

Phase contrast (PC) pulse sequences allow for the assessment of moving fluids in magnetic resonance imaging (MRI). Using volumetric data acquisition with multi-directional velocity encoding, complex hemodynamics in the heart and the adjacent great vessels can be assessed [1]. Resulting time-resolved volume datasets provide both the exact quantitative assessment of flow velocities and the intuitive visualization of flow phenomena using three-dimensional stream- and pathlines [2,3].

Numerous 4D flow MRI studies have demonstrated that the presence of vortex flow patterns in the heart chambers [4–6], the aorta [7], the carotid sinus [8], and pulmonary circulation [9] is physiological, but also relates to certain pathologies including aortic aneurysms [10–14], pulmonary hypertension [15], bicuspid aortic valves (BAV) [16,17], and congenital heart defects [18].

While physiological helices may help to stabilize flow, preserve energy, and protect vessels from atherosclerosis [7,19], pathologically altered vortical flow may have a number of detrimental effects. First, axial flow velocities are reduced by vortical flow, thus less blood is passing through a constant vessel section per unit time, resulting in a possible decrease of organ perfusion [20] or increase of cardiac work [21] *(reduction of blood current)*. Second, non-physiological vortex patterns may exert strain on the vessel wall, resulting in dilatation or in endothelial disorders [16,20–22] *(morphological alterations)*. Third, the velocity of blood platelets is reduced inside a vortex, possibly promoting blood clots or thrombosis *(rheological abnormalities)*.

First attempts of quantitative evaluation of vortical flow have been made. Hope et al. [11] employed a visual approach. Number of vortices, their start/end/peak frame, angle, and size were analyzed. However, such subjective measures might be inconvenient for clinical practice. The Helical Flow Index (HFI) was proposed as a more objective parameter measuring helical flow based on pathlines [23]. The average of local normalized helicity (LNH, equal to relative helicity *H*_*r*_ described below) was calculated along each pathline trajectory. To obtain a measure of global helicity, data of all pathlines were averaged. However, pathlines do not necessarily cover the entire aortic lumen and helicity quantification during diastole might be difficult. Bissell et al. [16] quantified rotational flow by use of circulation *Γ* [24], which is defined as the line integral of fluid velocity on the boundary of a closed surface (i.e., a vessel’s cross-section). Using Stokes’s theorem, it can be shown that *Γ* equals the integral of vorticity over the enclosed area. To reduce noise in the data, 5 time frames around peak systole were averaged for rotational flow measurements. A drawback of this approach is, however, that time-dependent information is lost. Bächler et al. [9] proposed a semi-automated approach by placing two-dimensional planes inside a vessel, exporting velocity values, and plotting absolute maximum vorticity values over time using a separate software tool. By this approach, however, important contextual and three-dimensional information is lost. Lorenz et al. [25] used equidistantly distributed 2D planes along the entire thoracic aorta to quantify altered blood flow helicity in bicuspid aortic valve (BAV) patients as compared to healthy volunteers. Helicity density *H*_*d*_ (being the dot product of the velocity and the correspondent vorticity vector) and relative helicity *H*_*r*_ (equaling the cosine of the angle between both vectors) were averaged over each analysis plane, parts of the aortic lumen, or periods of the heart cycle. Limitations of this approach include the insufficient extraction of vortices without any longitudinal flow component by the helicity parameter [18], information loss on minor counterrotating fluid components due to averaging [11,23], as well as omission of important three-dimensional information in the two-dimensional analysis. Recently, Elbaz et al. [6] proposed parameters for the quantification of left ventricular (LV) vortex ring formation during early and late diastole. The location and orientation of vortex rings (defined as λ_2_ isosurfaces) were characterized using normalized cylindrical coordinates relative to the shape of the left ventricle (i.e., circumferential, longitudinal, and radial coordinates; the ring’s orientation relative to the LV axis; and the circularity index defined as the ratio between the vortex’s short and long diameter). However, these parameters are not valid for anatomical regions other than the left ventricle and for vortical structures not equaling a vortex ring; in addition they do not provide insight into the strength or temporal evolution of the vortex. Three-dimensional vortex cores have been identified in previous studies, but have not been quantified [8,22,26,27].

The objective of the present work is to define quantitative metrics for objective characterization of complex vortical flow features (both two- and three-dimensionally), which can be calculated in a fully automated way. The metrics are applied to the analysis of the physiological vortex in the aortic arch during systole [7,10,13,22,25,28–31]. MR datasets acquired from 9 healthy subjects and 3 patients with dilated aortas are used to demonstrate the value of the method.

## Theory and Calculation

### 2.1 Terminology

The terms *vortex* and *helical flow* are not consistently defined in the literature. In medical studies, the term *helix* often describes fluid rotating around an axis while moving forward. The term *vortex* denotes rotating motion, where stream- or pathlines tend to curl back on themselves [22]. In the fluid dynamics domain, the terms *vortex* and *vortex core* are often used in a broader sense including helical movement [8,32–36]. Explanatory notes on the difficulty of an exact theoretical definition of the term *vortex* can be found in [32]. In this paper, the term *vortex* will be used in the wider definition of the fluid dynamics domain.

### 2.2 Vorticity

**Fig 1:**
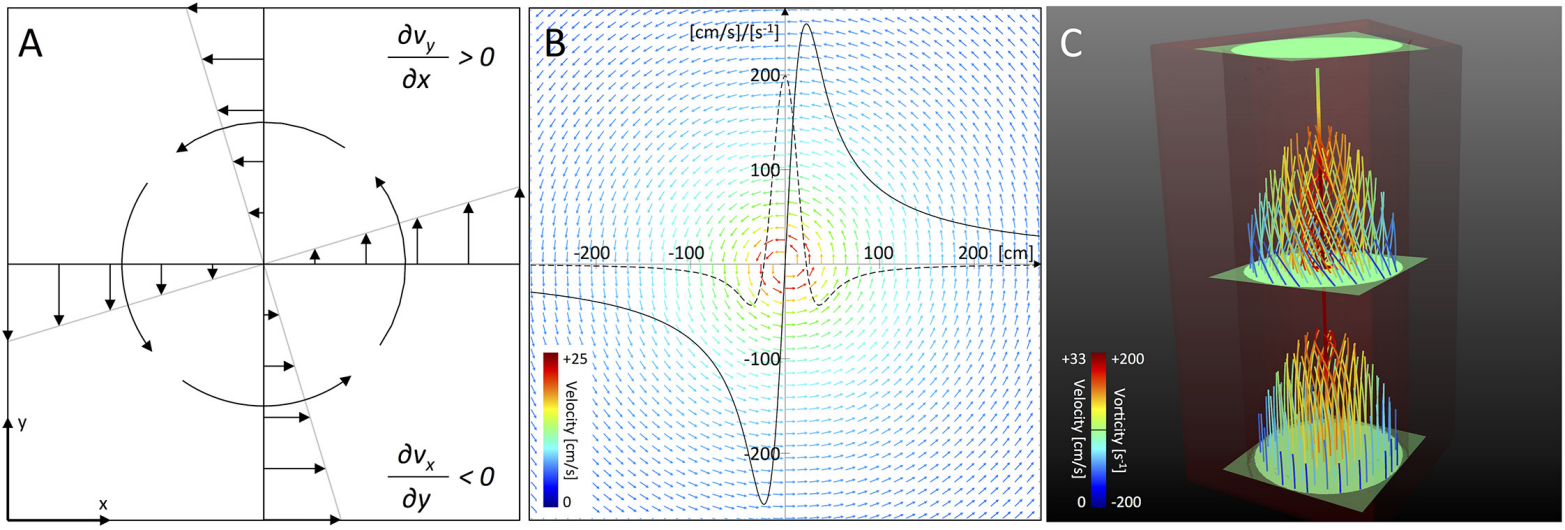
Vorticity Calculation. (A) Vorticity is defined as the curl of the velocity field (equal to (∂*v*_*y*_/∂*x*– ∂*v*_*x*_/∂*y*)*e*_*z*_ in the two-dimensional case), describing the tendency of a single fluid element to spin around its axis (as shown for a counter-clockwise rotating vortex; figure similar to [5]). (B) Illustration of the velocity field defined by the Lamb-Oseen equation, giving a theoretical approximation of a planar vortex. The solid line denotes the tangential velocity profile, while the dotted line depicts its first derivative. Note the derivative’s maximum in the vortex center due to the sudden shift of velocity values. (C) Artificial sample data superimposing a planar vortex on the parabolic velocity profile through a simple tube. Note the vortical movement of pathlines in the middle of the tube, intersecting ROIs exhibiting vorticity values, and the correctly identified vortex core.

To provide a basis for further methods outlined below, calculation of vorticity of the time-resolved three-dimensional vector field derived from 4D flow MRI measurements was implemented. The *vorticity ω* is defined as the curl of the velocity vector field $\vec{v}$ [37] (Fig 1A):

$$\vec{\omega }=\nabla \vec{v}$$

with

$$\nabla \vec{v}=\left(\frac{\partial }{\partial x},\frac{\partial }{\partial y},\frac{\partial }{\partial z}\right)\left(v_{x},v_{y},v_{z}\right)=\left(\frac{\partial v_{z}}{\partial y}-\frac{\partial v_{y}}{\partial z},\frac{\partial v_{x}}{\partial z}-\frac{\partial v_{z}}{\partial x},\frac{\partial v_{y}}{\partial x}-\frac{\partial v_{x}}{\partial y}\right).$$

Thus, a vorticity vector is given for every point–the norm of which describes the strength, while the direction represents the orientation of swirl. Positive values represent counter-clockwise (CCW) rotation, while negative values stand for clockwise (CW) fluid motion. The unit of vorticity is given as s^-1^.

In addition, the vorticity delivers information about the global form of a vortex (becoming maximum in the vortex’s center) due to the tangential velocity profile inside the vortex and its first derivative as shown in Fig 1B.

### 2.3 Lambda_2_

To facilitate further vortex characterization, a popular approach commonly referred to as the *λ*_*2*_ *-method* [38] was chosen. To define λ_2_, let $\vec{v}\left(x,y,z\right):={\left(v_{x},\;v_{y},v_{z}\right)}^{T}$ be the underlying velocity vector field. The velocity gradient or Jacobian of the velocity field is a 3x3 tensor containing information on how the velocity is changing in space [39]:

$$J_{v}=\left[\;\begin{array}{ccc} \frac{\partial v_{x}}{\partial x} & \frac{\partial v_{x}}{\partial y} & \frac{\partial v_{x}}{\partial z}\\ \frac{\partial v_{y}}{\partial x} & \frac{\partial v_{y}}{\partial y} & \frac{\partial v_{y}}{\partial z}\\ \frac{\partial v_{z}}{\partial x} & \frac{\partial v_{z}}{\partial y} & \frac{\partial v_{z}}{\partial z} \end{array}\;\right].$$

*J*_*v*_ can be decomposed into its symmetric part *S* and its antisymmetric part Ω. Physically, *S* describes the strain-rate tensor (containing both bulk and shear components), while Ω describes the rotation tensor (containing the rotational part of the flow) [38,39]:

$$S=\frac{1}{2}\left(J_{v}+J_{v}^{\;T}\right)\;\Omega =\frac{1}{2}\left(J_{v}-J_{v}^{\;T}\right).$$

Because the 3x3 matrix *S* ^*2*^ *+*Ω^*2*^ is real and symmetric, it has 3 real eigenvalues. Let λ_1_, λ_2_, and λ_3_ be the eigenvalues with λ_1_ ≤ λ_2_ ≤ λ_3._ According to Jeong and Hussain [38], a vortex can be defined as a region where two of the eigenvalues are negative, that is λ_2_ < 0. According to its definition, λ_2_ is a scalar quantity.

The main advantages of the method are its reliability in vortex identification [6,33,34,36,38] and its superiority in the context of 4D flow MRI [18]. Since derivatives are examined instead of velocity values, constant deltas added to the flow field cancel each other out, making the method Galilean invariant and applicable for time-varying flow fields [32,33]. Since only adjacent grid cells are involved in the calculation, it is also computationally inexpensive [32,33].

## Materials and Methods

### 3.1 Calculation of Vorticity Fields

While the vorticity of a differentiable vector field is defined infinitesimally, the spatial resolution of experimental MR data is limited. Multiple algorithms have been described for the precise computation of the vorticity field including [40,41]:

- first order central difference,
- 8-point-circulation method,
- higher order central difference.

**Fig 2:**
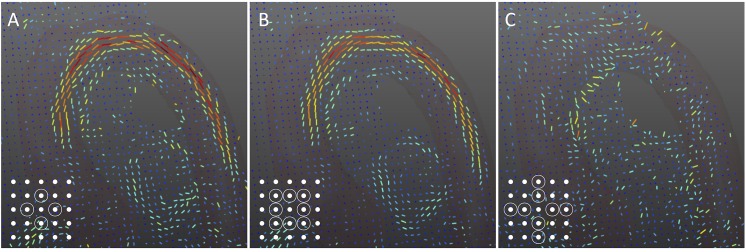
Algorithm Comparison. Vector fields resulting from three different algorithms for vorticity approximation: (A) first order central difference, (B) 8-point-circulation method, and (C) second order central difference. In the bottom left corner, the calculation scheme of each algorithm is given, illustrating adjacent data points that are taken into account for derivative calculation (schemes similar to [40]). While all three algorithms were adopted to a three-dimensional pattern for this work, 2D patterns are shown for simplicity.

Originally defined for two-dimensional vector fields, the algorithms were extended in a three-dimensional fashion and implemented for verification and comparison in our study (Fig 2). Resulting vector fields were rated by visual comparison. Evaluation criteria included noise, consistency of the vector field, and identification of vortex features.

### 3.2 2D: Region-of-Interest Based Analysis

**Fig 3:**
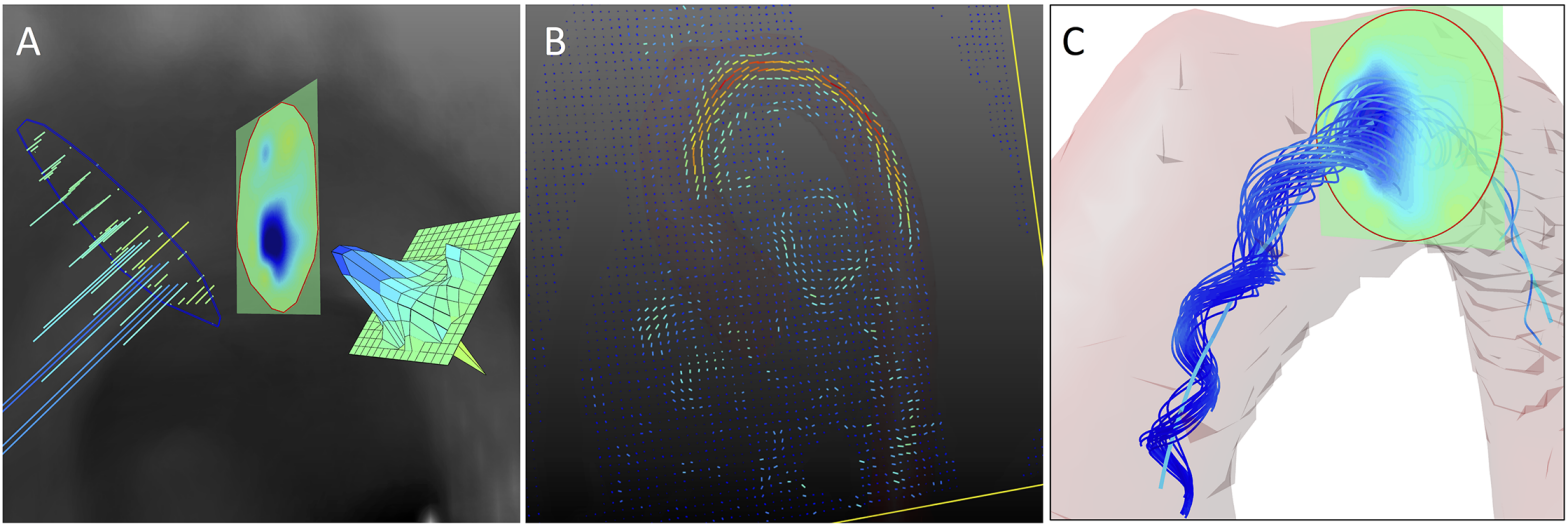
2D Visualization. (A) Visualization of vorticity values inside a ROI can be realized as a set of vectors, heatmap, or meshplot. Heatmap visualization lends itself to an intuitive vorticity analysis in its native three-dimensional surroundings, while vectors should be used to study the exact orientation of swirl. In this example, full vorticity vectors were limited to their through-plane component–correlating to swirl in the orientation of the ROI. (B) Two dimensional visualization of vorticity values as a color coded vector field, giving a first idea of areas with strong rotational flow. (C) A set of characteristic streamlines of the physiological vortex in the aortic arch, combined with an intersecting ROI displaying vorticity values and the automatically identified vortex core.

**Fig 4:**
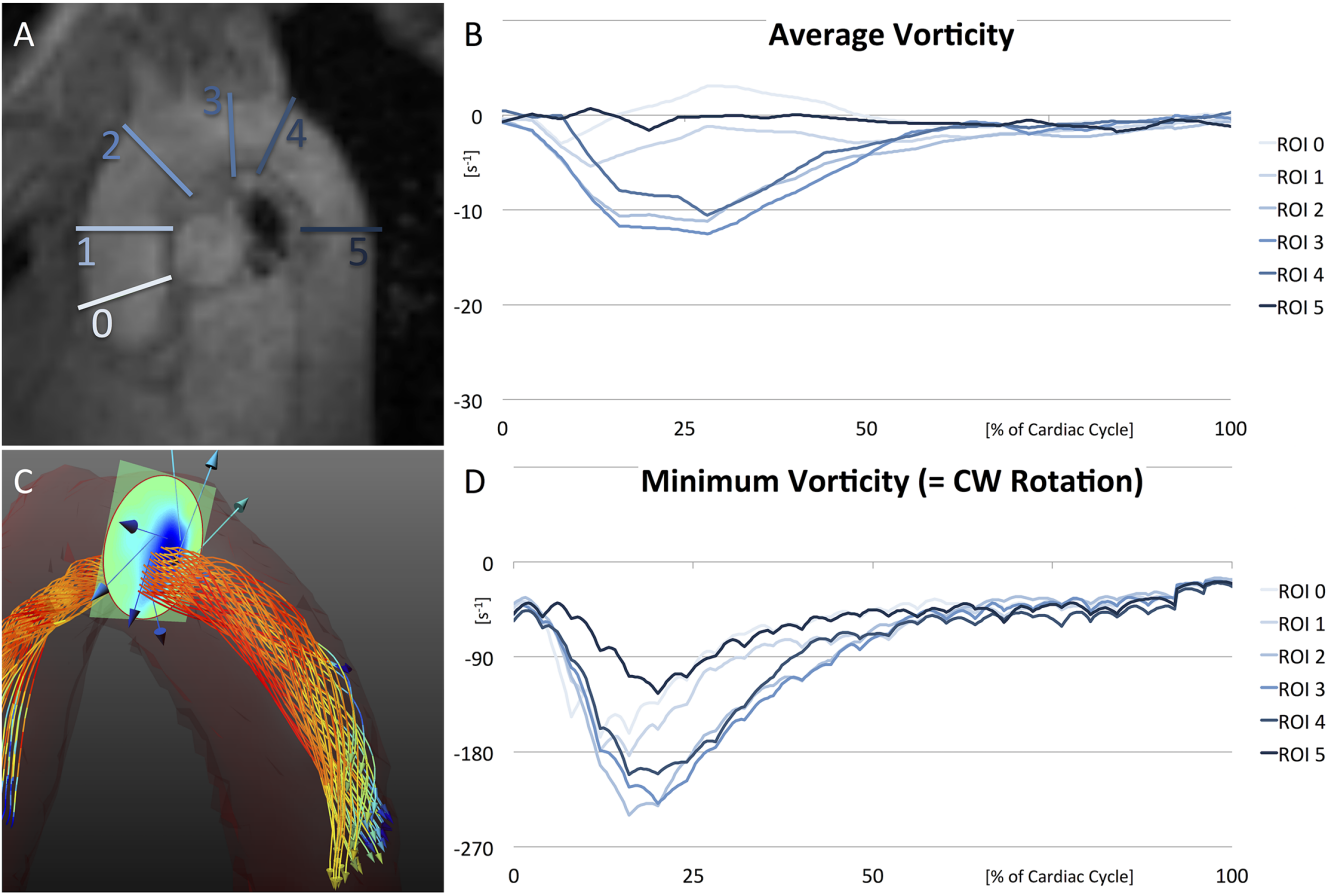
2D Quantification (summarized). (A) Set of 6 standard ROIs in the aorta, following standardized image interpretation guidelines [42,43]. (B) Average vorticity values of all ROIs plotted over time (averaged over all healthy subjects). (C) Physiological helix in the aortic arch; combined visualization of one ROI, in-plane velocity vectors, and pathlines within the segmented vessel. (D) Minimum vorticity values of all ROIs denoting clockwise rotation as the main rotational flow component (averaged over all healthy subjects). Note the early development of the vortex in ROIs 0 and 1 and its minimum in mid systole in the central ROIs 2, 3, and 4. Underlying data of the diagrams can be found in S1 Data.

**Fig 5:**
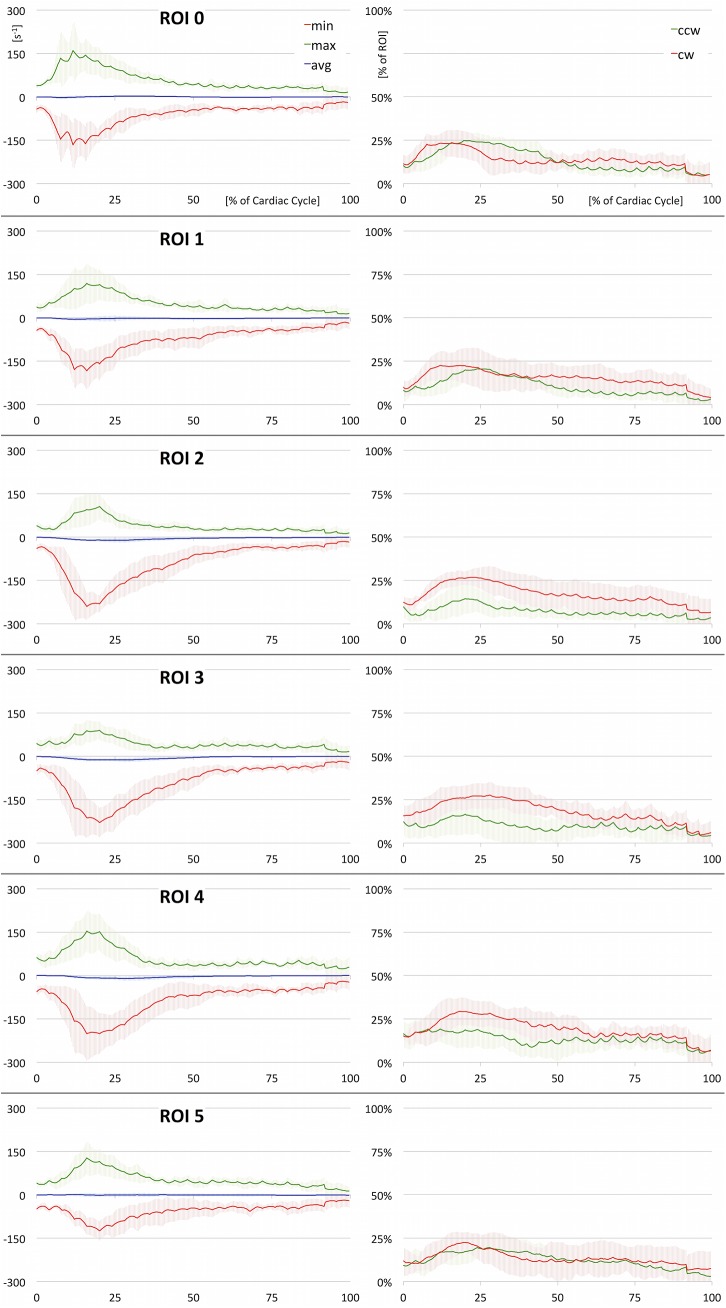
2D Quantification (full). Results of ROI based quantitative analysis (averaged over all healthy subjects). On the left, minimum, maximum, and average values of vorticity are plotted over time. These plots deliver insight into the vortex strength and rotational direction. On the right, the percentage of clockwise and counter-clockwise flow relative to the entire lumen is depicted. These plots deliver information on the amount of opposing rotational flow components. Underlying data of the diagrams is provided in S2 Data.

We aimed for an efficient integration of ROI based vortex analysis (Fig 3) yielding two-dimensional quantification of vortices (Figs 4 and 5). Therefore, a standardized set of 6 ROIs (Fig 4A) was defined for each dataset, 0: directly above the aortic bulb, 1: horizontal slice through the ascending aorta, 2: proximal to the brachiocephalic artery, 3: between the left common carotid artery and left subclavian artery, 4: distal to the left subclavian artery, 5: axial slice through the descending aorta at the level of ROI 1. All regions-of-interest were defined on cut planes perpendicular to the vessel lumen and restricted by the vessel wall. Positions were chosen according to standardized image interpretation guidelines [42,43] and slightly adjusted to fit into the specific context. For each ROI, a heatmap of time-resolved through-plane vorticity values was computed. The evolution of minimum, maximum, and average values throughout the cardiac cycle was plotted and analyzed (Figs 4 and 5). Since these values only contain information about vortex strength, the percentage of CW and CCW values exceeding an arbitrary threshold (set to 10 s^-1^ to suppress noise) was analyzed to estimate the amount of contrary rotational flow (Fig 5).

Resulting data of all healthy subjects were averaged for analysis; quantitative values are given as mean ± standard deviation. Patient data were analyzed individually to highlight pathologically altered flow.

### 3.3 3D: Vortex Core Analysis

Since a vortex has a spatial extent, it should also be studied in its native three-dimensional domain.

#### Visual exploration

To ease the understanding of flow characteristics, stream- and pathlines can be limited to a subset of lines already containing all relevant information, while irrelevant lines are abandoned to reduce visual distraction [44,45]. Multiple quality criteria for line extraction have been examined in previous studies with λ_2_ giving the best results for vortex identification [18]. In this project, characteristic sets of stream- and pathlines dependent on λ_2_ were defined to provide an instant overview of vortical flow features contained in the datasets (Fig 3C).

#### Vortex core detection

A vortex is intuitively understood as the swirling motion of fluid around a central region [32]. Multiple algorithms have been described to find the *core* of a vortex to accurately characterize its length, formation in space, and temporal evolution [32–36,38]. In the given scope of application, the vortex core algorithm needs to fulfill certain prerequisites [32]: (1) The core should be represented as a single line to enable length measurement. In addition, measuring the radial expansion of the vortex should be possible. (2) Due to the time-varying flow field, the method needs to be Galilean (Lagrangian) invariant. (3) For performance reasons, the number of grid cells involved in the computation should be minimized to a local area instead of examining the global vector field.

**Fig 6:**
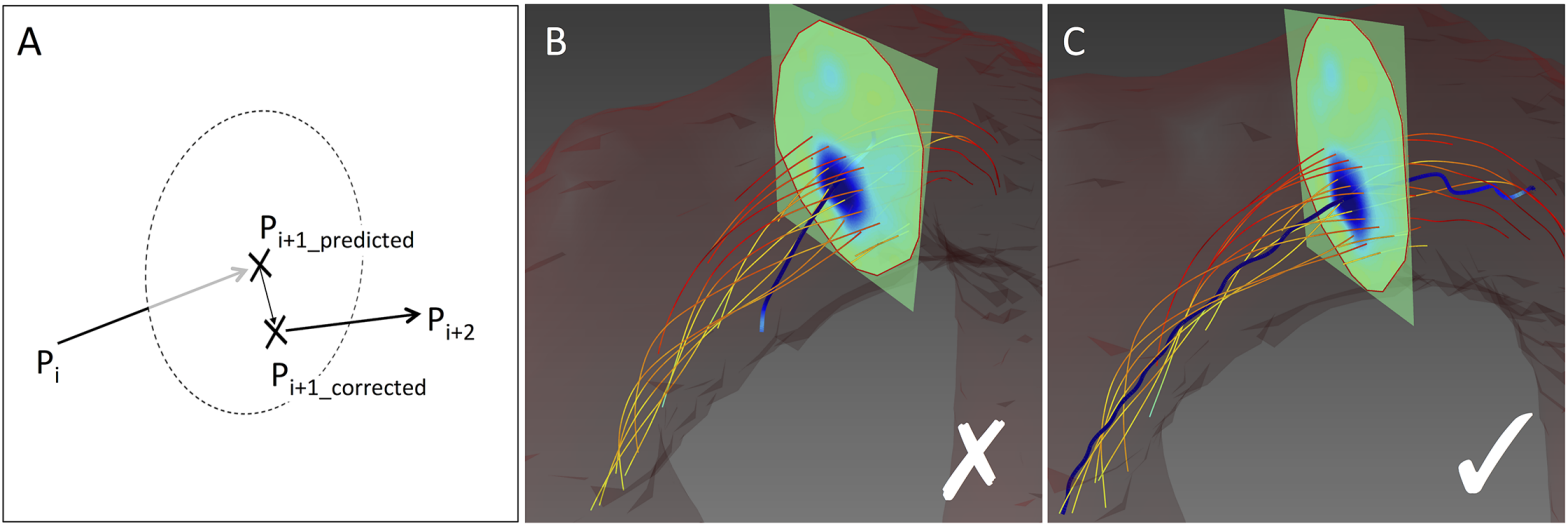
3D Vortex Core Detection. (A) Self-correcting approach of the vortex core identification algorithm. (B) An algorithm simply applying a streamline analysis to the vorticity field does not find the correct vortex core due to noise in the underlying MR velocity data. (C) With the predictor-corrector method, the core line is re-adjusted in every correction step so that single noisy data points do not compromise the rest of the vortex core identification process.

Therefore, an extension of the predictor-corrector method [35] was chosen for vortex core identification, since it conforms to all aforementioned pre-conditions. Assuming that a vortex reveals points of minimum pressure in its core, seed points *p*_*0*_ are defined as local pressure minima. In every step (Fig 6A), the algorithm elongates the vortex core by following the vorticity vector *ω*_*i*_ in the current point *p*_*i*_ (*prediction step*). The new position *p*_*i+1*_ is then corrected to the point of minimum pressure in a plane perpendicular to the vorticity vector *ω*_*i+1*_ (*correction step*). The core line is extended by repeating both steps until a predefined stopping criterion is reached.

The described method is robust due to its self correcting character (Fig 6B and 6C), but also has certain drawbacks: First, it does not work at low Reynolds numbers [33,38]. Second, pressure is hard to compute from a velocity field [33]. To overcome these issues, we used a method combining the predictor-corrector method with λ_2_, which is easy to compute from the flow field yet robust and effective for vortex identification. Instead of searching for the pressure minimum in the correction step, the vortex core is now corrected to the minimum λ_2_ value in the perpendicular plane [33].

Unlike the original suggestion by Banks and Singer [35], we applied a fourth order Runge-Kutta scheme [41] to extend the core line along the vorticity vector in the prediction step. In the correction step, we applied pattern search optimization [46] for finding the λ_2_ minimum rather than the originally proposed method of steepest descent [35]. This approach has the advantage of being derivative-free and resulted in more robust and faster convergence.

To quantify the cross section of the vortex, we radially sampled the vorticity field in the perpendicular plane in each correction step. Eight radial lines were tracked until |*ω*| fell under a given threshold (set to 150 s^-1^). The area *πr* ^*2*^ of a circle with radius *r* (with *r* equal to the average line length) was calculated as a measure for the vortex’s radial extent.

#### Vortex core quantification

The following three-dimensional parameters of the vortex core were recorded over time:

- *Vortex core length*. This value contains information about the longitudinal extent of the vortex.
- The *minimum*, *maximum*, and *average vorticity* throughout the course of the vortex core. These values hold information about the strength of the vortex.
- The *maximum cross section* of the vortex throughout its course. This measure informs about the maximum radial extent of the vortex at each time point.

Results of all healthy subjects were averaged for further analysis.

### 3.4 Implementation Specific Details

All described methods were implemented in C++ as a separate module inside the commercially available 4D flow software package GTFlow (GyroTools LLC, Zurich, Switzerland). For mathematical computation, the linear algebra template library Eigen (version 3.0.b2, eigen.tuxfamily.org) was used. For 3-dimensional visualization, Coin3D (version 3.1.3, www.coin3d.org) was employed. Coin3D is an OpenGL based graphics library compatible with the Open Inventor 2.1 API.

## Experimental

### 4.1 Sample Data

To evaluate the accuracy of the proposed techniques and to verify their implementation, artificial datasets were created. Results of all visualization and quantification methods analyzing these test data were compared to the mathematically exact solution. The test data contained a vortex superimposed on the laminar flow profile through a simple tube (Fig 1C).

Given a linear tube with a radial cross-section of radius *R*, the parabolic flow profile can be derived [47]:

$$v\left(r\right)=v_{m}\left(1-\frac{r^{2}}{R^{2}}\right)\;\text{and}\;v_{m}=-\frac{1}{2}\frac{R^{2}}{\mu }\frac{\Delta P}{\Delta x}$$

with *v*_*m*_ = maximum velocity, *∆P* = pressure difference, *∆x* = tube length, *μ* = viscosity, *r* = radius at point under consideration, and *R* = total tube radius. The Lamb-Oseen equation describes the temporal evolution of a planar vortex dependent on the fluid’s viscosity and was used for vorticity simulation in previous studies [40,48,49]. The tangential velocity equals:

$$v\left(r,t\right)=\frac{\Gamma }{2\pi r}\left(1-exp\left(\frac{-r^{2}}{r_{c}^{2}\left(t\right)}\right)\right)$$

with *r* and *μ* as defined above, $r_{c}\left(t\right)=\sqrt{4\mu t}$ = core radius of vortex, and *Γ* = circulation contained in the vortex. To define a smooth progression of the vortex, *v(r*,*t)* was multiplied by 0.5(cos(*πz* / *l*)+1) for *|z|≤l* with *l* = vortex length.

The first order central difference was used for vorticity calculation of the sample data, since both other methods (i.e., 8-point-circulation and higher order central difference) introduce smoothing [40], which is advantageous in noisy datasets, but adds imprecision to numerically exact data.

### 4.2 MR-Datasets

To demonstrate applicability of the newly defined methods to in-vivo data, a cohort of 9 healthy subjects was scanned. 4D flow MRI datasets covering the left-ventricular outflow tract, ascending, and descending aorta were acquired on a Philips 3T Achieva system (Philips Healthcare, Best, The Netherlands). Scan parameters of the respiratory navigated and retrospectively ECG triggered sequence were: FOV = 320x256x46 mm^3^, matrix = 160x160x20, SENSE reduction factor = 2, TR = 3.05–3.94 ms, flip angle = 5°, Venc_x,y_ = 200 cm/s, Venc_z_ = 100 cm/s, cardiac phases = 25, resulting scan resolution = 2.3x2.3x2.3 mm^3^. Next, 2 patients with mildly dilated ascending aortas were scanned (35.6 mm and 36.8 mm diameter of the proximal ascending aorta, respectively). Last, 1 patient with aortic aneurysm was scanned (49.2 mm diameter of the proximal ascending aorta). In this case, adapted scan parameters included: FOV = 350x280x75 mm^3^, matrix = 176x176x30, TR = 4.10 ms, cardiac phases = 20, Venc_x,z_ = 50 cm/s, Venc_y_ = 60 cm/s.

Vessels were segmented by applying a threshold on the velocity component product weighted by the magnitude image value. Cubic temporal interpolation of factor 10 was employed for refinement of limited resolution MR data. Cubic spatial interpolation of dynamic degree was applied at points reached during vortex core calculation.

### 4.3 Ethics Statement

All subjects gave written informed consent prior to MR scanning. The study complied with the Declaration of Helsinki and was approved by the ethics committee of the Canton of Zurich.

## Results

### 5.1 Calculation of Vorticity Fields

Vorticity fields in the aortic arch as computed by the three alternative algorithms are shown in Fig 2. Visual comparison indicated sufficiently accurate vorticity detection using the first order central difference (2A) and the 8-point-circulation method (2B). As can be seen in the figure, the latter method showed less vulnerability to noise in the evaluated dataset, resulting in a more consistent vorticity field. The second order central difference did not deliver reasonable results (2C).

### 5.2 Method Validation on the Basis of Sample Data

The exact knowledge of flow characteristics in the artificial sample dataset allowed validation of all described methods (Fig 1C). Characteristic stream- and pathlines clearly depicted the vortex in the linear tube. A ROI intersecting the tube showed the correct vorticity profile as expected from the definition of the Lamb-Oseen equation. Also the vortex core was correctly identified in the center of the tube. Numerical data were in good accordance with anticipated results for higher resolution test data (Table 1). Analysis of low resolution test data showed higher discrepancy relative to the mathematically exact results.

**Table 1:** Results of Method Validation. Comparison of measured values with the mathematically exact solution of the exemplarily defined sample dataset.

ROI		Exact Values	1 mm Resolution	±	0.1 mm Resolution	±
Max Vorticity	[s^-1^]	397.9	330.0	17 %	394.2	1 %
**CORE**						
Max Vorticity	[s^-1^]	397.9	343.8	14 %	381.4	4 %
Min Vorticity	[s^-1^]	50.0	50.1	0 %	50.0	0 %
Avg Vorticity	[s^-1^]	253.3	223.3	12 %	226.2	11 %
Length	[mm]	76.9	75.1	2 %	78.0	1 %

### 5.3 2D and 3D Vortex Visualization

**Fig 7:**
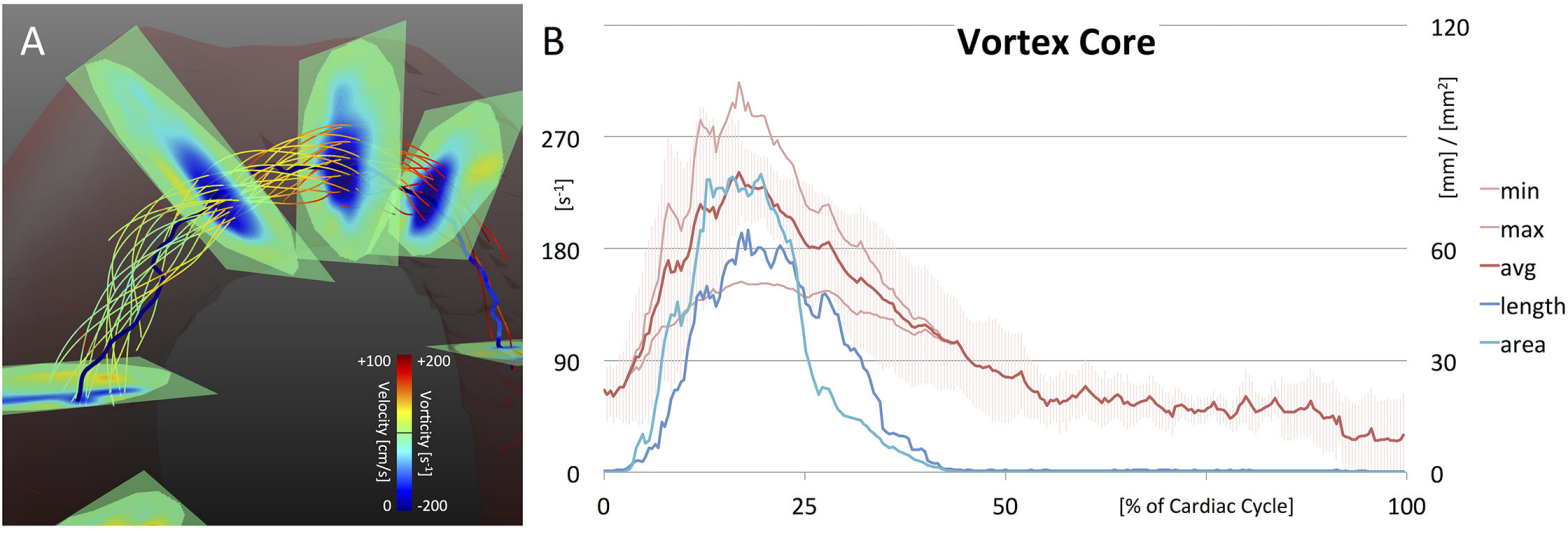
3D Quantification. (A) The resulting vortex core of one healthy subject. The core line is passing through points of minimum λ_2_. Note flow rotating around the core line. (B) Results of quantitative vortex core analysis, representing the vortex’s strength, elongation, and radial expansion (averaged over all healthy subjects). Underlying data of the diagram can be found in S3 Data.

The combined use of characteristic stream- or pathlines, vorticity or λ_2_ heatmaps inside the predefined ROIs, and automatic vortex core identification yielded intuitive three-dimensional visualization of the studied flow patterns (Figs 1C, 3C, 4C and 7A). Vortical flow features hidden in the multiplicity of stream- or pathlines could be easily identified and traced.

### 5.4 2D: Region-of-Interest Based Analysis

By comparing the temporal evolution of vorticity values in the 6 standardized ROIs, it was possible to estimate at which point in space and time a vortex developed, how it propagated, and where and when it faded out.

In Fig 4, average and minimum vorticity values (representing the main rotational flow component) of all standard ROIs are combined for direct comparison. The plots provide an overview of overall vortex progression in the aorta, but do not reveal areas with opposite rotation. Therefore, the temporal evolution of minimum, maximum, and average vorticity values as well as percentage of opposing flow components are separately illustrated for each ROI in Fig 5. In Table 2, minimum and maximum vorticity values of each ROI are listed with their corresponding time-point of occurrence.

**Table 2:** Results of 2D Quantification. For each of the standardized ROIs, minimum and maximum vorticity values (averaged over all healthy subjects) are listed with their standard deviation and time-point of occurrence (in % of cardiac cycle).

ROI	min [s^-1^]	SD	Time	max [s^-1^]	SD	Time
0	-166.4	± 86.4	12 %	160.1	± 102.0	12%
1	-183.6	± 65.3	16 %	118.8	± 67.5	16 %
2	-240.1	± 45.2	16 %	106.0	± 43.1	20 %
3	-229.2	± 50.0	20 %	89.9	± 30.7	20 %
4	-201.6	± 92.7	16 %	153.8	± 72.1	16 %
5	-125.0	± 33.2	20 %	127.1	± 57.6	16 %

*CW rotational flow* (denoted by negative values in Figs 4 and 5 and Table 2) developed during early systole. It could be observed first in ROI 0 and 1, which were located directly above the aortic bulb and in the ascending aorta, respectively (ROI 0: minimal vorticity = -166.4±86.4 s^-1^ at 12% of cardiac cycle and ROI 1: -183.6±65.3 s^-1^ at 16%). Thereupon, rotational flow propagated to the more distal ROIs 2, 3, and 4 with minimum values seen in mid systole (ROI 2: -240.1±45.2 s^-1^ at 16%, ROI 3: -229.2±50.0 s^-1^ at 20%, and ROI 4: -201.6±92.7 s^-1^ at 16%). The CW rotational flow diminished with overall low values during diastole. In the descending aorta, only minor rotational flow was present over all time points (ROI 5: -125.0±33.2 s^-1^ at 20%).

Results were different for *CCW rotational flow* (positive values in Fig 5 and Table 2). Again, vorticity increased during early systole, followed by maximum values in mid-systole and a final decrease in late systole. However, CCW rotational flow components were weaker during the entire heart cycle. They showed noticeable attenuation in the ascending aorta and proximal aortic arch with the progression of the right-handed helix (ROI 1: 118.8±67.5 s^-1^ at 16%, ROI 2: 106.0±43.1 s^-1^ at 20%, and ROI 3: 89.9±30.7 s^-1^ at 20%). Percentage plots of opposing flow components (Fig 5, right graphs) underline this finding (higher CW percentage of 27–29% compared to CCW percentage of 13–17% in ROIs 2, 3, and 4). Even though CCW rotational flow components are present over all time points, they did not lead to a fully developed vortex in the aorta.

### 5.5 3D: Vortex Core Analysis

Three-dimensional vortex core analysis is illustrated in Fig 7. The automatically identified vortex core followed the minimum λ_2_ values and was surrounded by the right-handed helical flow (7A). The core line exactly passed regions of minimum λ_2_ as can be seen for the intersected ROIs. The serrated nature of the core line was possibly caused by noise in the underlying MR velocity data. However, if gone astray, the core line was re-adjusted in every correction step of the identification algorithm so that single noisy data points did not compromise the rest of the vortex core identification process.

In Fig 7B, results of quantitative vortex core analysis are plotted. Vorticity strength increased in early systole and reached its maximum in mid systole (average vorticity = 241.2±30.7 s^-1^ at 17% of cardiac cycle). In diastole, the vortex strength decreased finally reaching overall low values (> 50% of cardiac cycle). Slightly delayed in the beginning, vortex elongation paralleled the strength values. The elongation reached its peak in mid systole (65.1±34.6 mm at 18%), before decreasing to low values in diastole. The vortex’s radial expansion slightly hurried ahead in early systole and declined faster after peaking in mid systole (80.1±48.8 mm^2^ at 20%).

### 5.6 Pathologically Altered Flow

**Fig 8:**
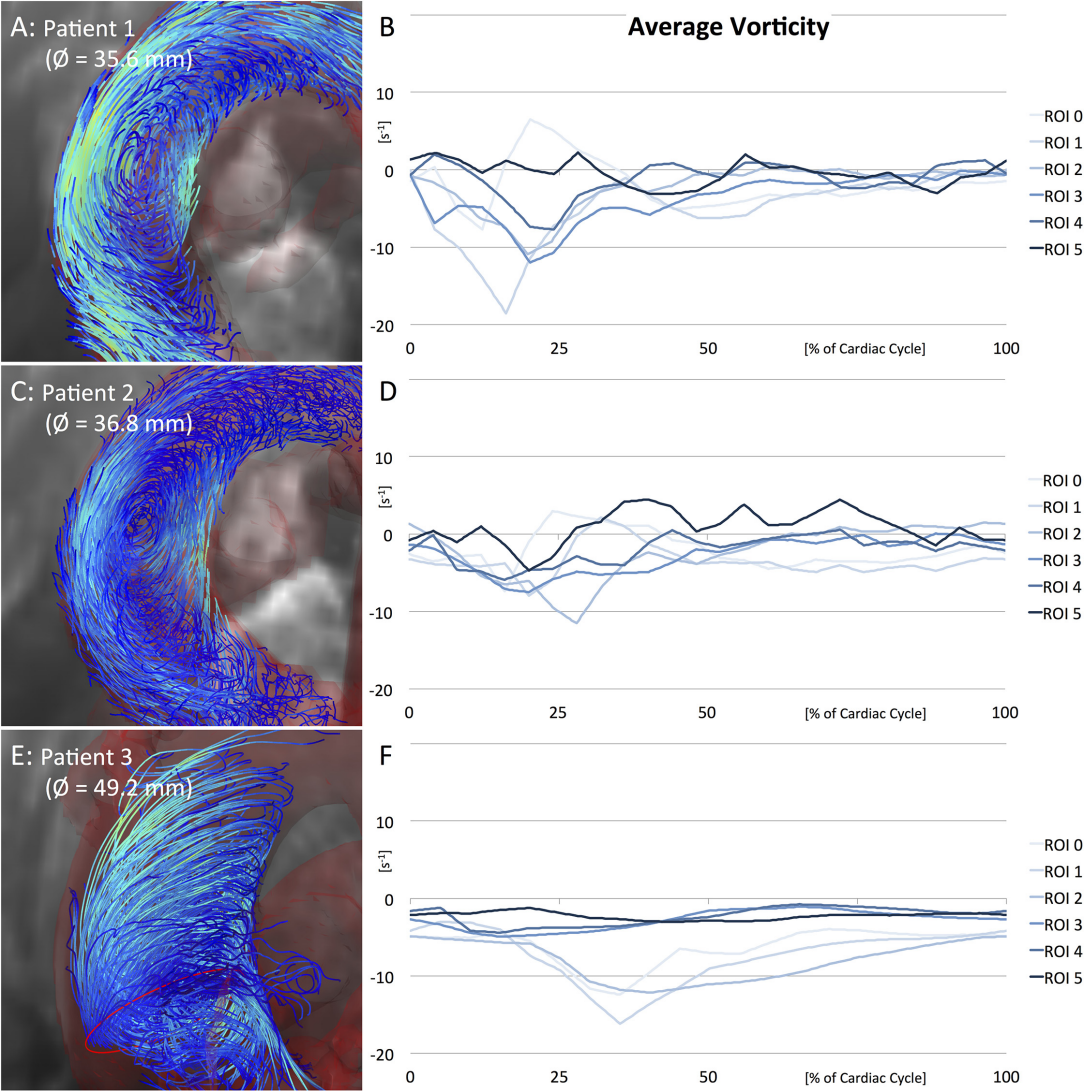
Pathologically Altered Flow. Pathlines and average vorticity plots of 2 patients with mildly dilated aortas (A/B and C/D) and 1 patient with aortic aneurysm (E/F). Note the heavily altered vortex flow in all three measurements. Physiological helical flow in the aortic arch completely diminishes in patient 3 (E/F). Underlying data of the diagrams is provided in S4 Data.

Pathologically altered vortex flow was observed in patient data. Fig 8 shows pathlines and average vorticity plots of the 6 standard ROIs for each case.

A comparison of all three patients indicated that changes in vortical flow got more pronounced as the aortic dilatation progressed (patient 1 < 2 < 3). In patient 1, the physiological helix through central ROIs was still visible (ROI 3: minimum of -115.8 s^-1^ at 24% of cardiac cycle), even though flow was considerably altered by a strong perpendicular swirl in the ascending aorta. In patient 2, alterations were more pronounced and the physiological helix further vanished (ROI 3: -108.0 s^-1^ at 20%). In patient 3, swirling flow developed late in mid/end-systole close to the aortic bulb (ROI 1: -229.1 s^-1^ at 30%), and no physiological helix was found in the aortic arch (ROI 3: -44.1 s^-1^ at 10%).

A vortex core line was identified in the ascending aorta of all patients by the vortex core detection algorithm, but results were not fully satisfactory and stable.

## Discussion

In our study, we implemented different visualization techniques to allow for intuitive exploration of vortical flow features of time-resolved velocity vector fields. 2D and 3D quantitative metrics for the evaluation of vortex flow were introduced. After validation against artificial sample data, the techniques were successfully applied to quantify physiological vortex flow in the aortic arch of healthy volunteers and to identify pathological flow alterations in 3 cases of aortic dilatation.

Flow patterns in the aortic arch have been examined in a number of previous studies. Kilner et al. [7] were the first to describe a predominant right-handed helical flow in the upper aortic arch in mid to late systole in 9 out of 10 subjects, while flow patterns in the descending aorta varied greatly among subjects. By now, the right-handed helical flow is a well acknowledged physiological flow feature found in most subjects [10,13,22,25,28–31], especially in younger individuals with crook-shaped arch geometry [29]. In accordance with this we found a right-handed vortex arising in the ascending aorta early in systole and propagating to the aortic arch with peak vorticity values reached in mid systole. While also counter-clockwise rotational flow components were measurable, no fully left-handed vortex developed in the aorta.

Multiple studies have examined the influence of age [29–31], geometry [29], and vascular pathologies [20,30] on aortic flow characteristics. Already minor geometric variations in the aortic size and shape were found to considerably alter blood flow characteristics as compared to healthy subjects [20]. In bicuspid aortic valve disease, increased right-handed helical flow has been described to be the most common flow alteration in the ascending aorta, possibly leading to increased rotational wall shear stress and progressive aortic dilatation [16,25]. Nested helical flow was found at peak systole [17]. In ascending aortic aneurysms, pathological findings included helical and vortical flow [10–12,14] as well as prolonged retrograde flow [11]. Comparing aortic aneurysms in more detail, a large variety of pathological vortex flow patterns has been reported depending on the individual cause of dilatation [13]. In our small cohort of patients the severity of hemodynamic aberrations seemed to be gradually related to the degree of aortic dilatation. While in patients with mild aortic ectasia physiological flow features were still discernable, the patient with aortic aneurysm showed pronounced alterations of flow patterns and vorticity values.

All these studies suggest an important role of flow alterations in the pathophysiology of aortic disease. The complexity of flow patterns, however, makes it difficult to reliably identify flow features that determine disease progression and prognosis just by means of visual analysis. In order to advance the knowledge of potentially detrimental effects of certain hemodynamic changes a more systematic analysis is needed, which was the aim of our study.

We therefore compared three algorithms for vorticity calculation, differing in the set of surrounding points that are taken into account for computation. The 8-point-circulation method gave the best subjective results in our specific case, being in good accordance with the literature [40]. Poor results of the second order central difference scheme may have been caused by the low resolution of MRI data; instead of adding valuable information, more distant points may hamper precise derivative computation. Extensive theoretical comparison and uncertainty analysis of the alternative algorithms can be found in [40].

In our study, all methods were validated in a set of artificially defined sample data. Arising deviations from mathematically exact solutions were carefully inspected. We found that the implementation of our methods worked correctly with the given data. However, since values of the tangential velocity profile shift quickly inside a vortex center (Fig 1B), data of low spatial resolution tend to miss relevant velocity values and thereby compromise precise vorticity calculation. These findings indicate that highly resolved data play a decisive role in accurate flow pattern analysis.

With the visualization techniques implemented in our study we were able to consistently visualize vortical flow in the aorta of all scanned individuals. The combination of characteristic stream- or pathlines, standardized ROIs, vorticity/λ_2_ heatmaps, and automatically identified vortex core lines allowed identification of regions with vortical flow.

In addition to a more consistent visual exploration, objective quantitative metrics are needed to better characterize flow pattern changes occurring under pathological conditions. Limiting the examination of flow data to certain, carefully chosen regions-of-interest is a well-known technique widely used in the field of 4D flow MRI [50]. It is used for both the quantification of blood flow velocities and the qualitative description of flow patterns. Even though parts of the vector field might not be fully examined, ROIs help the investigator to abstract from the overwhelming complexity of the flow field and to concentrate on certain areas containing most relevant information. In this study, we transferred this commonly used technique to vorticity quantification: Two-dimensional quantitative analysis in consecutive, standardized ROIs was useful for following vortex paths over time. By these means, we were able to consistently detect and quantify the typical development of right handed helical flow in the aortic arch in all our healthy subjects, fully accordant with findings by Lorenz et al. [25]

Three-dimensional quantification was realized by analyzing the elongation, strength, and radial expansion of an automatically detected vortex core. A quantitative description of flow characteristics in the aortic arch observed in 9 healthy subjects was performed with results being in good consistency with physiological flow qualitatively described in the literature [10,13,22,28–31]. The three-dimensional algorithm led to less robust results in patient data, which is associated with lower velocity-to-noise ratio (VNR) and low vorticity values. In Fig 1, the Lamb-Oseen equation was introduced as a theoretical approximation of the velocity profile inside a vortex. Accordingly, the vorticity reaches maximum values inside the vortex center due to maximum shift of velocity. If overall velocity is low or the vortex broadens, vorticity values decrease, resulting in a less stable output of the identification algorithm.

### 6.1 Study Limitations

While the focus of our study was the definition and validation of techniques for visualizing and quantifying swirling blood in 4D flow MRI data, the number of healthy subjects and patients enrolled in the study remained small. Even though the measures of vorticity proved stable in all healthy volunteers and were in good agreement with findings in the literature, a larger cohort is needed to establish reference values for vorticity in the healthy aorta. Moreover, further studies with larger numbers of patients are needed to test whether alterations of vortical flow in the aorta are related to disease severity or may even help to predict disease progression.

In our study the methods were applied to a single anatomical region only. Future work should include in-depth analysis of different vascular territories in order to elucidate the role that vortical flow may play in other anatomical regions (e.g., heart chambers, pulmonary circulation, or carotid arteries) and pathologies (e.g., pulmonary hypertension or congenital heart defects).

## Conclusions

In the present work, two- and three-dimensional metrics for the quantification of vortical blood flow in the thoracic aorta have been proposed and validated. While this study can serve as a proof of concept, future work should assess whether and how these techniques can help to diagnose and grade flow related pathologies and their prognosis.

## Supporting Information

S1 Data Underlying data of Fig 4. (XLSX)

S2 Data Underlying data of Fig 5. (XLSX)

S3 Data Underlying data of Fig 7. (XLSX)

S4 Data Underlying data of Fig 8. (XLSX)

## References

[1] Wigström L, Sjöqvist L, Wranne B. Temporally resolved 3D phase-contrast imaging. Magn Reson Med Off J Soc Magn Reson Med Soc Magn Reson Med. 1996;36: 800–803. 10.1002/mrm.1910360521 8916033

[2] Wigström L, Ebbers T, Fyrenius A, Karlsson M, Engvall J, Wranne B, et al Particle trace visualization of intracardiac flow using time-resolved 3D phase contrast MRI. Magn Reson Med Off J Soc Magn Reson Med Soc Magn Reson Med. 1999;41: 793–799. 10.1002/(sici)1522-2594(199904)41:4<793::aid-mrm19>3.0.co;2-2 10332856

[3] Buonocore MH. Visualizing blood flow patterns using streamlines, arrows, and particle paths. Magn Reson Med. 1998;40: 210–226. 10.1002/mrm.1910400207 9702703

[4] Wong KKL, Kelso RM, Worthley SG, Sanders P, Mazumdar J, Abbott D. Cardiac flow analysis applied to phase contrast magnetic resonance imaging of the heart. Ann Biomed Eng. 2009;37: 1495–1515. 10.1007/s10439-009-9709-y 19466548

[5] Wong KKL, Tu J, Kelso RM, Worthley SG, Sanders P, Mazumdar J, et al Cardiac flow component analysis. Med Eng Phys. 2010;32: 174–188. 10.1016/j.medengphy.2009.11.007 20022796

[6] Elbaz MSM, Calkoen EE, Westenberg JJM, Lelieveldt BPF, Roest AAW, Geest RJ van der. Vortex flow during early and late left ventricular filling in normal subjects: quantitative characterization using retrospectively-gated 4D flow cardiovascular magnetic resonance and three-dimensional vortex core analysis. J Cardiovasc Magn Reson. 2014;16: 78 10.1186/s12968-014-0078-9 25270083 PMC4177574

[7] Kilner PJ, Yang GZ, Mohiaddin RH, Firmin DN, Longmore DB. Helical and retrograde secondary flow patterns in the aortic arch studied by three-directional magnetic resonance velocity mapping. Circulation. 1993;88: 2235–2247. 10.1161/01.CIR.88.5.2235 8222118

[8] Stalder A, Frydrychowicz A, Harloff A, Yang Q, Bock J, Henning J, et al. Vortex core detection and visualization using 4D flow-sensitive MRI. Proceedings 18th Scientific Meeting. Stockholm; 2010.

[9] Bächler P, Pinochet N, Sotelo J, Crelier G, Irarrazaval P, Tejos C, et al Assessment of normal flow patterns in the pulmonary circulation by using 4D magnetic resonance velocity mapping. Magn Reson Imaging. 2013;31: 178–188. 10.1016/j.mri.2012.06.036 22898700

[10] Markl M, Draney MT, Hope MD, Levin JM, Chan FP, Alley MT, et al Time-resolved 3-dimensional velocity mapping in the thoracic aorta: visualization of 3-directional blood flow patterns in healthy volunteers and patients. J Comput Assist Tomogr. 2004;28: 459–468. 15232376 10.1097/00004728-200407000-00005

[11] Hope TA, Markl M, Wigström L, Alley MT, Miller DC, Herfkens RJ. Comparison of flow patterns in ascending aortic aneurysms and volunteers using four-dimensional magnetic resonance velocity mapping. J Magn Reson Imaging JMRI. 2007;26: 1471–1479. 10.1002/jmri.21082 17968892

[12] Hope TA, Herfkens RJ. Imaging of the thoracic aorta with time-resolved three-dimensional phase-contrast MRI: a review. Semin Thorac Cardiovasc Surg. 2008;20: 358–364. 10.1053/j.semtcvs.2008.11.013 19251177

[13] Weigang E, Kari FA, Beyersdorf F, Luehr M, Etz CD, Frydrychowicz A, et al Flow-sensitive four-dimensional magnetic resonance imaging: flow patterns in ascending aortic aneurysms. Eur J Cardio-Thorac Surg Off J Eur Assoc Cardio-Thorac Surg. 2008;34: 11–16. 10.1016/j.ejcts.2008.03.047 18515137

[14] Bürk J, Blanke P, Stankovic Z, Barker A, Russe M, Geiger J, et al Evaluation of 3D blood flow patterns and wall shear stress in the normal and dilated thoracic aorta using flow-sensitive 4D CMR. J Cardiovasc Magn Reson. 2012;14: 84 10.1186/1532-429X-14-84 23237187 PMC3534249

[15] Reiter G, Reiter U, Kovacs G, Kainz B, Schmidt K, Maier R, et al Magnetic Resonance–Derived 3-Dimensional Blood Flow Patterns in the Main Pulmonary Artery as a Marker of Pulmonary Hypertension and a Measure of Elevated Mean Pulmonary Arterial Pressure. Circ Cardiovasc Imaging. 2008;1: 23–30. 10.1161/CIRCIMAGING.108.780247 19808511

[16] Bissell MM, Hess AT, Biasiolli L, Glaze SJ, Loudon M, Pitcher A, et al Aortic Dilation in Bicuspid Aortic Valve Disease: Flow Pattern Is a Major Contributor and Differs with Valve Fusion Type. Circ Cardiovasc Imaging. 2013; CIRCIMAGING.113.000528. 10.1161/CIRCIMAGING.113.000528 23771987 PMC3859916

[17] Hope MD, Hope TA, Meadows AK, Ordovas KG, Urbania TH, Alley MT, et al Bicuspid aortic valve: four-dimensional MR evaluation of ascending aortic systolic flow patterns. Radiology. 2010;255: 53–61. 10.1148/radiol.09091437 20308444

[18] Kohler B, Gasteiger R, Preim U, Theisel H, Gutberlet M, Preim B. Semi-Automatic Vortex Extraction in 4D PC-MRI Cardiac Blood Flow Data using Line Predicates. IEEE Trans Vis Comput Graph. 2013;19: 2773–2782. 10.1109/TVCG.2013.189 24051844

[19] Liu X, Pu F, Fan Y, Deng X, Li D, Li S. A numerical study on the flow of blood and the transport of LDL in the human aorta: the physiological significance of the helical flow in the aortic arch. Am J Physiol Heart Circ Physiol. 2009;297: H163–170. 10.1152/ajpheart.00266.2009 19429823

[20] Frydrychowicz A, Harloff A, Jung B, Zaitsev M, Weigang E, Bley TA, et al Time-resolved, 3-dimensional magnetic resonance flow analysis at 3 T: visualization of normal and pathological aortic vascular hemodynamics. J Comput Assist Tomogr. 2007;31: 9–15. 10.1097/01.rct.0000232918.45158.c9 17259827

[21] Bogren HG, Mohiaddin RH, Yang GZ, Kilner PJ, Firmin DN. Magnetic resonance velocity vector mapping of blood flow in thoracic aortic aneurysms and grafts. J Thorac Cardiovasc Surg. 1995;110: 704–714. 10.1016/S0022-5223(95)70102-8 7564437

[22] Markl M, Kilner PJ, Ebbers T. Comprehensive 4D velocity mapping of the heart and great vessels by cardiovascular magnetic resonance. J Cardiovasc Magn Reson. 2011;13: 7 10.1186/1532-429X-13-7 21235751 PMC3025879

[23] Morbiducci U, Ponzini R, Rizzo G, Cadioli M, Esposito A, Cobelli FD, et al In Vivo Quantification of Helical Blood Flow in Human Aorta by Time-Resolved Three-Dimensional Cine Phase Contrast Magnetic Resonance Imaging. Ann Biomed Eng. 2009;37: 516–531. 10.1007/s10439-008-9609-6 19142728

[24] Hess AT, Bissell MM, Glaze SJ, Pitcher A, Myerson S, Neubauer S, et al Evaluation of Circulation, Γ, as a quantifying metric in 4D flow MRI. J Cardiovasc Magn Reson. 2013;15: E36 10.1186/1532-429X-15-S1-E36

[25] Lorenz R, Bock J, Barker AJ, von Knobelsdorff-Brenkenhoff F, Wallis W, Korvink JG, et al 4D flow magnetic resonance imaging in bicuspid aortic valve disease demonstrates altered distribution of aortic blood flow helicity. Magn Reson Med. 2014;71: 1542–1553. 10.1002/mrm.24802 23716466 PMC3778148

[26] Meckel S, Stalder AF, Santini F, Radü E-W, Rüfenacht DA, Markl M, et al In vivo visualization and analysis of 3-D hemodynamics in cerebral aneurysms with flow-sensitized 4-D MR imaging at 3 T. Neuroradiology. 2008;50: 473–484. 10.1007/s00234-008-0367-9 18350286

[27] Heiberg E, Ebbers T, Wigstrom L, Karlsson M. Three-dimensional flow characterization using vector pattern matching. IEEE Trans Vis Comput Graph. 2003;9: 313–319. 10.1109/TVCG.2003.1207439

[28] Frydrychowicz A, Berger A, Russe MF, Stalder AF, Harloff A, Dittrich S, et al Time-resolved magnetic resonance angiography and flow-sensitive 4-dimensional magnetic resonance imaging at 3 Tesla for blood flow and wall shear stress analysis. J Thorac Cardiovasc Surg. 2008;136: 400–407. 10.1016/j.jtcvs.2008.02.062 18692649

[29] Frydrychowicz A, Berger A, Munoz Del Rio A, Russe MF, Bock J, Harloff A, et al Interdependencies of aortic arch secondary flow patterns, geometry, and age analysed by 4-dimensional phase contrast magnetic resonance imaging at 3 Tesla. Eur Radiol. 2012;22: 1122–1130. 10.1007/s00330-011-2353-6 22207269

[30] Bogren HG, Mohiaddin RH, Kilner PJ, Jimenez-Borreguero LJ, Yang GZ, Firmin DN. Blood flow patterns in the thoracic aorta studied with three-directional MR velocity mapping: the effects of age and coronary artery disease. J Magn Reson Imaging JMRI. 1997;7: 784–793. 9307902 10.1002/jmri.1880070504

[31] Bogren HG, Buonocore MH. 4D magnetic resonance velocity mapping of blood flow patterns in the aorta in young vs. elderly normal subjects. J Magn Reson Imaging. 1999;10: 861–869. 10.1002/(SICI)1522-2586(199911)10:5<861::AID-JMRI35>3.0.CO;2-E 10548800

[32] Jiang M, Machiraju R, Thompson D. Detection and visualization of vortices The Visualization Handbook. Academic Press; 2005 pp. 295–309.

[33] Stegmaier S, Rist U, Ertl T. Opening the Can of Worms: An Exploration Tool for Vortical Flows. IEEE; 2005 pp. 59–59. 10.1109/VIS.2005.74

[34] Sahner J, Weinkauf T, Hege H-C. Galilean Invariant Extraction and Iconic Representation of Vortex Core Lines. 2005; Available: http://130.203.133.150/viewdoc/similar;jsessionid=C0EAB663543F0F7FE5BC45F7FBE483D2?doi=10.1.1.131.3207&type=ab

[35] Banks D, Singer BA. Vortex Tubes in Turbulent Flows: Identification, Representation, Reconstruction. Society Press; 1994 pp. 132–139.

[36] Schafhitzel T, Vollrath JE, Gois JP, Weiskopf D, Castelo A, Ertl T. Topology-Preserving λ2-based Vortex Core Line Detection for Flow Visualization. Comput Graph Forum. 2008;27: 1023–1030. 10.1111/j.1467-8659.2008.01238.x

[37] Majda AJ, Bertozzi AL. Vorticity and Incompressible Flow. Cambridge University Press; 2002.

[38] Jeong J, Hussain F. On the identification of a vortex. J Fluid Mech. 1995;285: 69–94. 10.1017/S0022112095000462

[39] Haimes R, Kenwright D. On the Velocity Gradient Tensor and Fluid Feature Extraction. 1999.

[40] Luff JD, Drouillard T, Rompage AM, Linne MA, Hertzberg JR. Experimental uncertainties associated with particle image velocimetry (PIV) based vorticity algorithms. Exp Fluids. 1999;26: 36–54. 10.1007/s003480050263

[41] Chapra SC, Canale RP. Numerical methods for engineers Boston: McGraw-Hill Higher Education; 2010.

[42] Schulz-Menger J, Bluemke DA, Bremerich J, Flamm SD, Fogel MA, Friedrich MG, et al Standardized image interpretation and post processing in cardiovascular magnetic resonance: Society for Cardiovascular Magnetic Resonance (SCMR) Board of Trustees Task Force on Standardized Post Processing. J Cardiovasc Magn Reson. 2013;15: 35 10.1186/1532-429X-15-35 23634753 PMC3695769

[43] Hiratzka LF, Bakris GL, Beckman JA, Bersin RM, Carr VF, Casey DE Jr, et al 2010 ACCF/AHA/AATS/ACR/ASA/SCA/SCAI/SIR/STS/SVM Guidelines for the Diagnosis and Management of Patients With Thoracic Aortic Disease. J Am Coll Cardiol. 2010;55: e27–e129. 10.1016/j.jacc.2010.02.015 20359588

[44] Born S, Pfeifle M, Markl M, Scheuermann G. Visual 4D MRI blood flow analysis with line predicates. 2012 IEEE Pacific Visualization Symposium (PacificVis). 2012. pp. 105–112. 10.1109/PacificVis.2012.6183580

[45] Born S, Pfeifle M, Markl M, Gutberlet M, Scheuermann G. Visual Analysis of Cardiac 4D MRI Blood Flow Using Line Predicates. IEEE Trans Vis Comput Graph. 2013;19: 900–912. 10.1109/TVCG.2012.318 23559505

[46] Dolan E, Lewis R, Torczon V. On the Local Convergence of Pattern Search. SIAM J Optim. 2003;14: 567–583. 10.1137/S1052623400374495

[47] Rogers DF. Laminar Flow Analysis. Cambridge University Press; 1992.

[48] Ruan X, Song X, Yamamoto F. Direct measurement of the vorticity field in digital particle images. Exp Fluids. 2001;30: 696–704. 10.1007/s003480000249

[49] Fouras A, Soria J. Accuracy of out-of-plane vorticity measurements derived from in-plane velocity field data. Exp Fluids. 1998;25: 409–430. 10.1007/s003480050248

[50] Markl M, Frydrychowicz A, Kozerke S, Hope M, Wieben O. 4D flow MRI. J Magn Reson Imaging. 2012;36: 1015–1036. 10.1002/jmri.23632 23090914

